# Combining Phylogenetic and Syntenic Analyses for Understanding the Evolution of TCP ECE Genes in Eudicots

**DOI:** 10.1371/journal.pone.0074803

**Published:** 2013-09-03

**Authors:** Hélène L. Citerne, Martine Le Guilloux, Julie Sannier, Sophie Nadot, Catherine Damerval

**Affiliations:** 1 Plant Genetics Research Unit, National Centre of Scientific Research, Paris-South University, National Institute of Agronomic Research, Gif-sur-yvette, France; 2 The Ecology, Systematics and Evolution Laboratory, Paris-South University, National Centre of Scientific Research –AgroParisTech, Orsay, France; Instituto de Biología Molecular y Celular de Plantas, Spain

## Abstract

TCP ECE genes encode transcription factors which have received much attention for their repeated recruitment in the control of floral symmetry in core eudicots, and more recently in monocots. Major duplications of TCP ECE genes have been described in core eudicots, but the evolutionary history of this gene family is unknown in basal eudicots. Reconstructing the phylogeny of ECE genes in basal eudicots will help set a framework for understanding the functional evolution of these genes. TCP ECE genes were sequenced in all major lineages of basal eudicots and *Gunnera* which belongs to the sister clade to all other core eudicots. We show that in these lineages they have a complex evolutionary history with repeated duplications. We estimate the timing of the two major duplications already identified in the core eudicots within a timeframe before the divergence of *Gunnera* and after the divergence of Proteales. We also use a synteny-based approach to examine the extent to which the expansion of TCP ECE genes in diverse eudicot lineages may be due to genome-wide duplications. The three major core-eudicot specific clades share a number of collinear genes, and their common evolutionary history may have originated at the γ event. Genomic comparisons in *Arabidopsis thaliana* and *Solanum* *lycopersicum* highlight their separate polyploid origin, with syntenic fragments with and without TCP ECE genes showing differential gene loss and genomic rearrangements. Comparison between recently available genomes from two basal eudicots *Aquilegia* *coerulea* and *Nelumbo* *nucifera* suggests that the two TCP ECE paralogs in these species are also derived from large-scale duplications. TCP ECE loci from basal eudicots share many features with the three main core eudicot loci, and allow us to infer the makeup of the ancestral eudicot locus.

## Introduction

Morphological innovations affecting reproductive structures have played a crucial role in the evolutionary success of flowering plants. Major shifts in floral morphology occurred during the evolution of the eudicots, the largest group of flowering plants with over 75% of extant species [1,2]. Two major groups of eudicots can be identified: a grade of early-diverging lineages (thereafter referred to as basal eudicots) comprising the Ranunculales, Sabiaceae, Proteales, Buxales and Trochodendrales (a total of ~4,650 species in 17 families), and the derived core eudicot clade, with over 200,000 species [2]. The emergence of the core eudicots (or more precisely the Pentapetalae comprising all core eudicots excluding Gunnerales [2,3]) coincides with the fixation of a pentamerous, whorled organization of the flower and a bipartite perianth (reviewed in [1,4]). By contrast, basal eudicots, like basal angiosperms, have variable floral structures with labile phyllotaxis (whorled and/or spiral), dimerous or trimerous (or rarely tetramerous or pentamerous) organ arrangement, and frequently an undifferentiated perianth, Ranunculales being a notable exception [1,5].

The fixation of the number of floral parts in the Pentapetalae is considered to have enabled the repeated evolution of floral elaborations such as synorganization and bilateral floral symmetry (zygomorphy) [6,7]. Zygomorphy can be described as the unequal placement or development of floral organs, most frequently the corolla and androecium, along a single axis. This axis is usually vertical from the inflorescence apex (adaxial or dorsal side) to the subtending bract (abaxial or ventral side). Zygomorphy is believed to have a function in pollinator attraction and specificity (reviewed in [8]), and has been associated with increased speciation rates [9]. It is estimated that zygomorphic flowers have evolved at least 42 times independently from radially symmetric ancestors in the core eudicots [10]. Although zygomorphy is considered to be rare in basal eudicots [6], it has evolved independently in Ranunculaceae, Papaveraceae, Menispermaceae, Sabiaceae and Proteaceae [6,10,11].

In core eudicots, the genetic control of zygomorphy has been found to involve the asymmetric, most frequently adaxial expression of homologs of *CYCLOIDEA* (*CYC*). The involvement of *CYC*-like genes in the control of floral symmetry has been shown or strongly implied in both asterids (Veronicaceae [12-16], Gesneriaceae [17,18], Asteraceae [19-25], Caprifoliaceae [26]), and rosids (Fabaceae [27-30], Brassicaceae [31,32], Malpighiaceae [33]). It has been suggested that the pattern of expression of *CYC*-like genes in the radially symmetric ancestor of rosids and asterids may also have been asymmetric on the adaxial side of the floral meristem prior to organogenesis, enabling the repeated recruitment of these genes in lineages which have evolved zygomorphic flowers independently [34]. In addition, recent work in monocots suggests that *CYC*-like genes are also implicated in the control of floral symmetry, determining organ differentiation within the perianth [35-37] and stamen abortion [36]. In basal eudicots, *CYC*-like gene expression has been described in flowers of Papaveraceae, and although no correlation of asymmetric expression with zygomorphy was observed at early developmental stages [38,39], later expression in the outer petals and nectaries in Fumarioideae was correlated with asymmetric growth along the transverse plane of the flower [40].

There is mounting evidence that the core eudicot radiation is preceded by a whole genome triplication (known as the γ event [41]) that is believed to have contributed to an increased complexity in gene interactions and the evolution of novel gene functions underlying phenotypic changes [42-45]. Duplication and triplication of major gene families prior to the divergence of core eudicots have been described for floral developmental genes, primarily from the MADS-box gene family ( [45] and references therein), and also the *DIVARICATA*-like gene family [46], which includes the *DIV* gene implicated in the determination of ventral identity in zygomorphic *Antirrhinum* *majus* flowers [47,48]. Two major gene duplications have also been described prior to the divergence of the core eudicots within the ECE clade of the TCP gene family, to which *CYC* belongs [49]. The plant-specific TCP gene family encodes transcription factors implicated in cell cycling and growth [50,51]. These genes are characterized by a distinctive basic helix-loop-helix domain, the TCP domain, whose structure defines two classes (I and II) within this gene family [50]. Among class II genes, members of the ECE clade also have an 18-20 residue arginine-rich motif (the R domain) and a relatively conserved glutamic acid – cysteine – glutamic acid (ECE) motif between the TCP and R domains. In addition to *CYC*, the best characterized member of the ECE clade is *TEOSINTE BRANCHED1* (*TB1*) which controls axillary branching and stamen development in maize and related grasses [52,53]. Because of the complex history of gene duplication both in grasses and core eudicots, these two genes are not orthologs*. CYC* and all other *CYC*-like genes implicated in the control of floral symmetry belong to the same clade in core eudicots (*CYC2*) [49]. The function of the genes from the two other clades is mostly unknown, although in *Arabidopsis thaliana*, *BRC1* (*TCP18*, *CYC1* clade) and to a lesser extent *BRC2* (*TCP12*, *CYC3* clade) control axillary bud development similarly to *TB1* [54].

Basal eudicots are frequently under-represented in studies of developmental genes, even though they diversified at a crucial point in angiosperm history. The diversity of TCP ECE genes in this grade is not known. A single copy was described in *Aquilegia* *alpina* (Ranunculaceae) [49], whereas two copies were found in Papaveraceae [38,39]. This study characterizes for the first time TCP ECE genes in all major basal eudicot lineages and in the early-diverging core eudicot *Gunnera* *tinctoria* , and reconstructs the phylogeny of ECE genes in eudicots in the light of these new sequences. In addition to phylogenetic reconstruction, we compare genome segments containing the TCP ECE genes from core eudicots (rosid and asterid) and two basal eudicots, to better understand the nature of the duplication events in this gene family.

## Materials and Methods

### DNA extraction, genomic fragment amplification and sequencing

Genomic DNA was extracted from leaf material from 13 taxa (12 representatives of the major lineages of basal eudicots and one representative of Gunneraceae (sample list and phylogeny given in Figure S1)), following a cetyl-trimethyl-ammonium-bromide (CTAB) method [55]. Genomic fragments were amplified by nested PCR using multiple degenerate primer sets (primers from [39], and this study (primer sequences given in Table S1)) designed to bind to conserved regions of the TCP and R domains. Cloned PCR products (pGEM-T Easy, Promega) were sequenced by Genoscreen (Lille, France). An average of 29 clones was sequenced from 2-4 nested primer combinations for each taxon. DNA sequences for each taxon were analyzed with BioEdit v.7.0.5.3 [56]. TCP genes were identified by the presence of the characteristic amino acid sequence of the TCP domain. Copy number was determined for each taxon by comparing sequence divergence between clones; clones considered to be of the same type were ~99-100% identical.

To minimize the impact of missing data on phylogenetic inference, the complete sequence of the TCP and R domains for each TCP ECE copy identified in this study was obtained by inverse PCR [57]. Sequences were verified by direct amplification (primer sequences given in Table S1). Sequences were deposited in GenBank at the National Centre for Biotechnology Information Database (http://www.ncbi.nlm.nih.gov) (accession numbers are given in Table S2).

### Phylogenetic analyses

In the first instance, fifty seven TCP ECE nucleotide sequences from 14 taxa belonging to all major eudicot clades and 3 monocot species were compiled in BioEdit v7.0.5.3 [56] (accession numbers given in Table S2). In addition to the selected sequences from this study, sampling consisted of species with characterized *CYC*-like genes: *Antirrhinum* *majus* (Plantaginaceae) (*CYC* [12], *DICH* [13], *AmTCP1, AmTCP5*), *Arabidopsis thaliana* (Brassicaceae) (*TCP1, TCP12, TCP18* [50]), *Helianthus annuus* (*CYC1a-b, CYC2a-e, CYC3a-c* [58]), *Chelidonium* *majus* (Papaveraceae (*CmCyL1, CmCyL2*[39]), and the basal-most monocot *Acorus* *calamus* (Acoraceae) (*TB1a, TB1b*[33]), and species with sequenced genomes 1) core eudicots : *Vitis vinifera* (grapevine; Vitaceae), *Populus* *trichocarpa* (poplar; Salicaceae), *Carica papaya* (papaya; Caricaceae), *Prunus persica* (peach; Rosaceae), *Solanum* *lycopersicum* (tomato; Solanaceae); basal eudicots: *Aquilegia* *coerulea* (Ranunculaceae); monocots: *Sorghum* *bicolor* and *Oryza sativajaponica* (Poaceae) (draft genome sequences explored by TBLASTN searches using the CoGe platform (http://genomevolution.org/CoGe)). Unambiguous alignment, done manually on translated amino acid sequences, was possible only in the TCP (177 bp), ECE (21 bp), and R (57 bp) domains. Phylogenetic analyses were carried out using maximum likelihood (ML) with PhyML 3.0 [59,60] and Bayesian inference implementing the Markov Chain Monte Carlo (MCMC) algorithm with MrBayes v3.1.2 [61]. The model of DNA substitution GTR + I + Γ selected by the Akaike Information Criterion using Modeltest v3.8 [62] was specified in PhyML and MrBayes. For the ML analysis, tree improvement was carried out by Subtree Pruning and Regrafting (SPR) and Nearest-Neighbor Interchange (NNI). Branch support was obtained by approximate likelihood-ratio test with the Shimodaira-Hasegawa (SH)-like approach [60] and is given as a percentage. For Bayesian inference, data was partitioned by codon position allowing variable substitution rates. Two independent analyses (nruns=2) of 4 chains (3 heated) were run simultaneously for 20 million generations, sampling every 1,000 generation. Burnin was estimated at 5 million generations (discarding the first 5,000 sampled trees). Majority rule consensus trees, summarizing topology and branch lengths of the sampled trees and posterior clade probability (PP, given as percentages), were obtained with MrBayes.

To help clarify the evolutionary history of TCP ECE in basal eudicots and early-diverging core eudicots, analyses with additional aligned characters were carried out with sequences from 14 basal eudicot species, *Gunnera* *tinctoria* , and three rosid species (*P. persica*, *P* *. trichocarpa* , and the early-diverging rosid *V. vinifera*). Sequence alignment of the variable region between the TCP and R domains was carried out with MUSCLE [63] with default parameters followed by manual adjustments in BioEdit v.7.0.5.3 [56]. Phylogenetic analyses were carried out as above. Gene duplication history was investigated by automatic tree reconciliation using NOTUNG 2.6 [64] from the gene tree (obtained with PhyML) containing TCP ECE sequences from *A* *. coerulea* , *Nelumbo* *nucifera* , *Platanus* *orientalis* , *Tetracentron* *sinense* , *Buxus* *sempervirens* , *G* *. tinctoria* , *V. vinifera*, and *P* *. trichocarpa* . To determine whether one of the duplications resulting in the *CYC1*, *CYC2* and *CYC3* clades may have predated the divergence of eudicots, we tested alternative topologies of the gene tree used for tree reconciliation. The baseml program from the PAML package v.4.4 [65] was used to calculate site-wise loglikelihoods for the set of trees compared. To take into account the coding nature of the sequences, a HKY model of evolution partitioned according to codon position (option Mgene=4) was used, following the recommendation of Yang [66]. The software CONSEL v 0.1i [67] was then used to perform the Approximately Unbiased test (AU [68]) and the weighted Shimodaira and Hasegawa test (WSH [69]).

### Synteny analysis

Genome synteny between three rosids *Arabidopsis thaliana* (NCBI v1), *Prunus persica* (JGI v1), *Vitis vinifera* (NCBI v3), one asterid *Solanum* *lycopersicum* (SGN v2.40), and two basal eudicots *Aquilegia* *coerulea* (JGI v1) and *Nelumbo* *nucifera* (NCBI v2) was detected using the SynFind program, and visualized using GeVo, (Genome Evolution analysis tool) from the CoGe platform (http://genomevolution.org/CoGe/). Separate searches were performed using the three TCP ECE genes from *P. persica* genome as queries. Gene identities were verified by BLAST homology searches. Locus-based genome exploration was also carried out using the Plant Genome Duplication Database (PGDD) (http://chibba.agtec.uga.edu/duplication/index/locus) [70].

## Results

### TCP ECE copy number in basal eudicots and phylogenetic analyses

One to three TCP ECE copies were isolated in the 13 species examined, with all but *Nandina* *domestica* and *Epimedium* *alpinum* having multiple copies. A *CINCINNATA*-like gene (belonging to a different clade of class II TCP genes) was also amplified with the same degenerate primers in *Akebia* *quinata* , *N* *. domestica* , and *Nelumbo* *nucifera* (GenBank accession numbers given in Table S2). BLAST searches on the recently available *N* *. nucifera* genome confirmed the two copies of TCP ECE genes isolated here by PCR ( *Nelumbo* *nucifera* 1= NNU_012730-RA, *Nelumbo* *nucifera* 2 = NNU_001168-RA).

### Phylogenetic analyses

**Figure 1:**
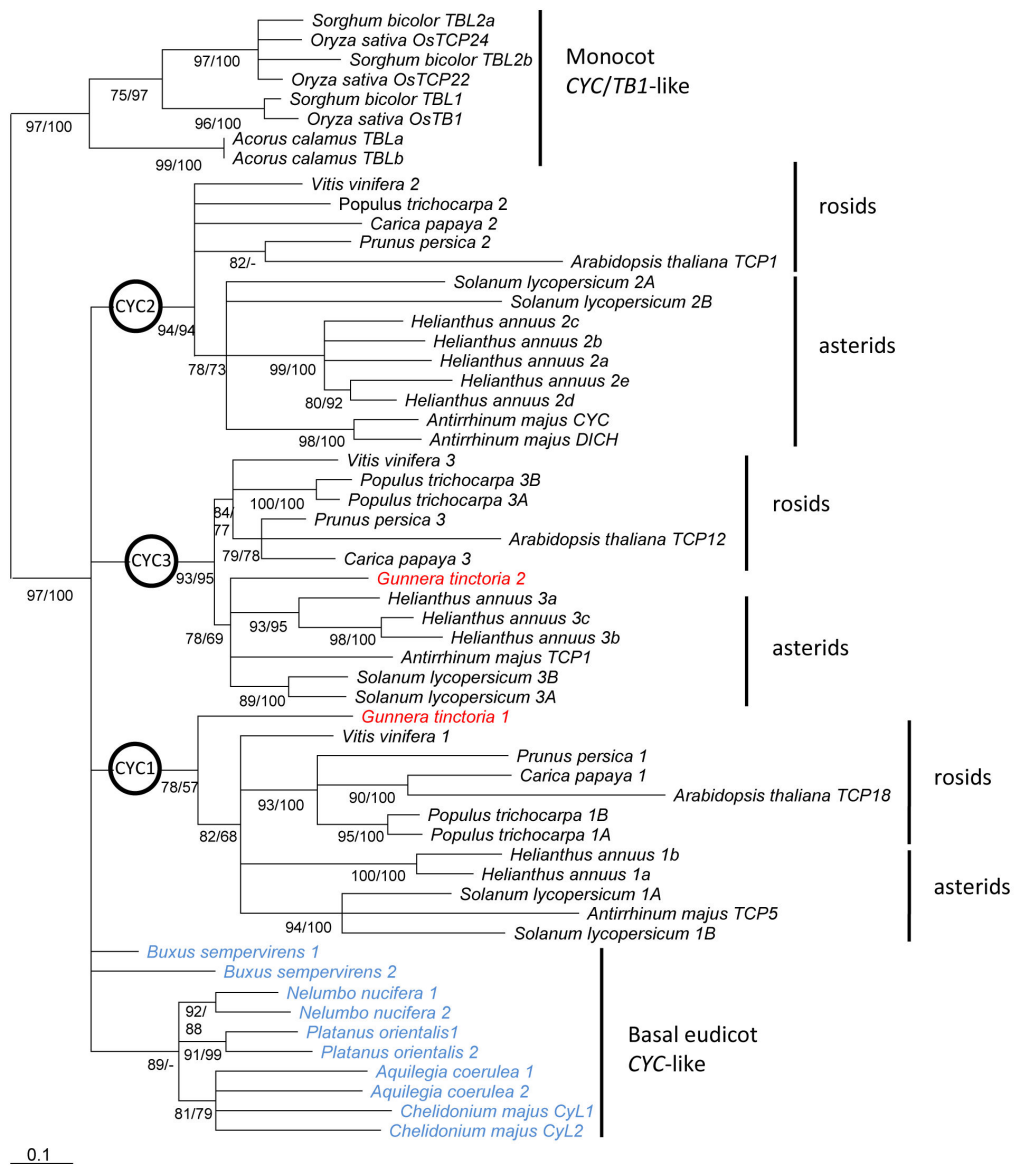
TCP ECE gene phylogeny in eudicots, rooted on monocot sequences. Phylogeny was inferred by ML analysis of the nucleotide sequences of the TCP, ECE and R domains (249 characters). Clades with ≥75% Shimodaira-Hasegawa (SH)-like support are shown; SH values are given below each branch; posterior probabilities (PPs) from the Bayesian analysis of the same dataset are given after. Sequence names of basal eudicots are in blue, and of *Gunnera* *tinctoria* in red.

Relationships among TCP ECE genes from monocots, basal eudicots and core eudicots were inferred from the conserved TCP, ECE and R regions. Both ML and Bayesian analyses showed that sequences from eudicots formed a well-supported clade (100% posterior probability (PP)/ 97% Shimodaira-Hagesawa-like support (SH)) (Figure 1). Deep relationships between basal and core eudicot sequences were unresolved. The *CYC1*, *CYC2* and *CYC3* clades identified in [49] were recovered, comprising asterid and rosid sequences as well as one copy from the early-diverging core eudicot *Gunnera* *tinctoria* in the *CYC1* and *CYC3* clades (Figure 1). Sequences from newly available core eudicot genomes (e.g. peach: *Prunus persica*, tomato: *Solanum* *lycopersicum* ) fit into the three characterized groups of TCP ECE genes, with two copies found in tomato for each group. One of the six copies of TCP ECE genes in *S* *. lycopersicum* , 2B, has a stop codon inserted before the R domain and appears to form a truncated protein that may be in the process of pseudogenization.

**Figure 2:**
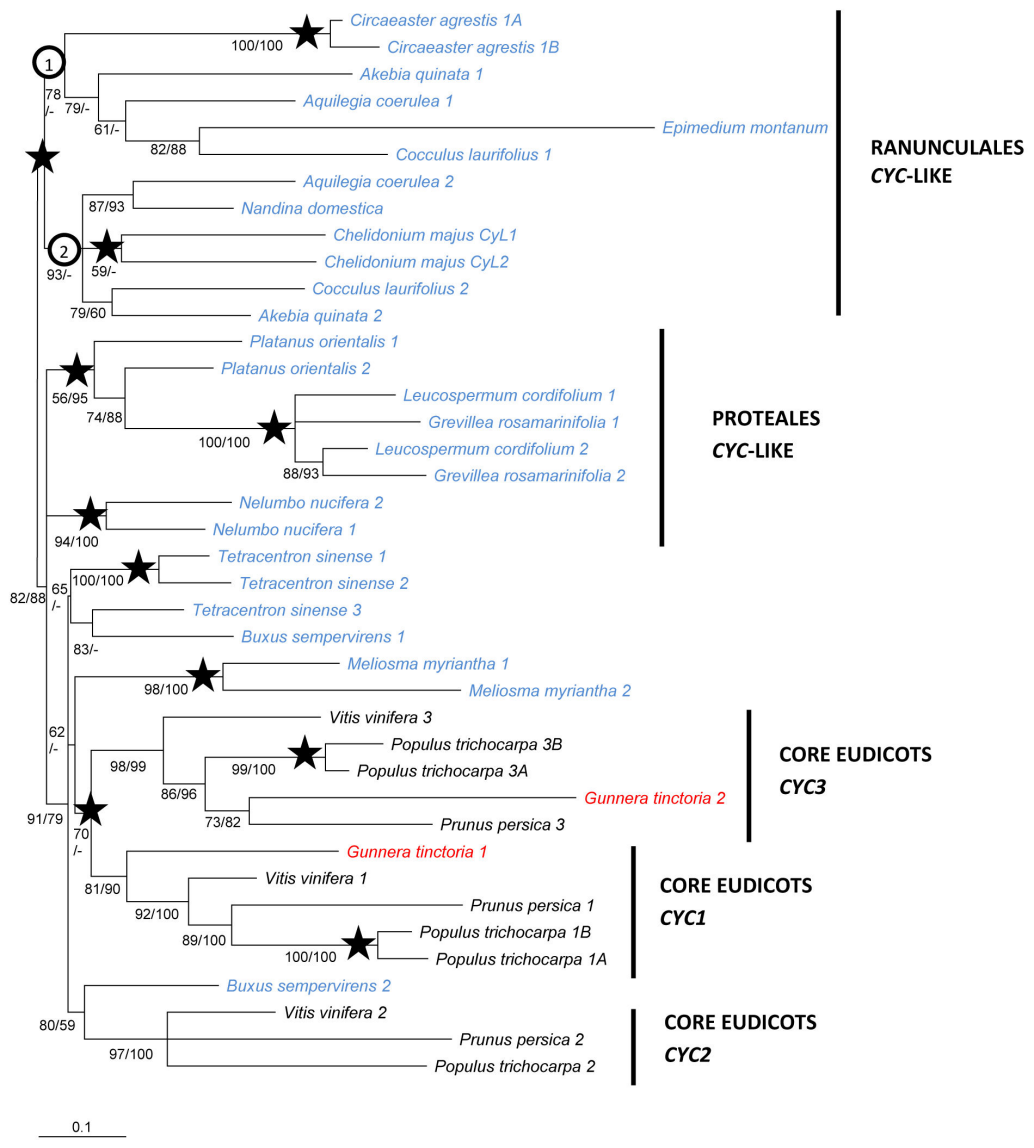
TCP ECE gene phylogeny in basal and core eudicots. Phylogeny was inferred by ML analysis of the aligned nucleotide sequences between, and including, the TCP and R domains (339 characters). Clades with ≥50% Shimodaira-Hasegawa (SH)-like support are shown. SH values are given below or to the left of each branch; posterior probabilities (PPs) from the Bayesian analysis of the same dataset are given after. Stars show resolved duplication events inferred from this tree. Sequence names are color-coded; basal eudicots: blue, *Gunnera* *tinctoria* : red, Pentapetalae: black.

Focusing on basal eudicot sequences (Figure 2), a number of well-supported lineage-specific paralogous clades were found, for example in *N* *. nucifera* (Nelumbonaceae), Proteaceae ( *Leucospermum* *cordifolium* (Proteoideae) and *Grevillea* *rosmarinifolia* (Grevilleoideae)), *Meliosma* *myriantha* (Sabiaceae), and *Tetracentron* *sinense* (Trochodendraceae) for copies 1 and 2. Other duplication events were unresolved as in *Buxus* *sempervirens* (Buxaceae) and *Tetracentron* *sinense* copies 1/2 and 3). In Ranunculales, the ML analysis recovered two clades (with 78% and 93% SH respectively, PP<50% in Bayesian analysis), each containing one copy from Ranunculaceae, Menispermaceae, and Lardizabalaceae. Only one copy was found in the two representatives of Berberidaceae, the sister family to Ranunculaceae, and these belonged to different clades (clade 1 for *E* *. alpinum* , and clade 2 for *N* *. domestica* ). The two copies identified in *Circaeaster* *agrestis* (Circaeasteraceae) were highly similar paralogs (94.8% identity) belonging to clade 1. In Papaveraceae, the two copies were found nested within clade 2, suggesting that the paralog from clade 1 may have been lost, and a lineage-specific duplication occurred in clade 2.

Despite the addition of more variable sequence data between the TCP and R domains in the data matrix, the evolutionary history of TCP ECE genes preceding the divergence of the core eudicots was not clearly resolved (Figure 2). Both ML and Bayesian analyses supported the placement of the core eudicot *CYC1*, *CYC2*, *CYC3* clades in a monophyletic group also comprising *T* *. sinense* , *M* *. myriantha* and *B* *. sempervirens* sequences (79% PP, 91% SH) (Figure 2). Reconciliation of the TCP ECE gene tree (where sequences from core eudicots, *T* *. sinense* and *B* *. sempervirens* form a monophyletic group (93% SH)) with the species tree suggested that the duplication events resulting in the three core eudicot *CYC* clades occurred after the divergence of Proteales, and before the divergence of *G* *. tinctoria* , but could not exclude *T* *. sinense* and *B* *. sempervirens* from these duplication events (Figure S2). Tests of alternative topologies excluded a duplication event prior to the divergence of the eudicots although the tree with the *CYC1* clade as sister to all other sequences belonged to the set of trees with 5% confidence with a probability of 0.24 (AU test) (Table S3).

### Genome synteny around TCP ECE genes in basal and core eudicots

Comparison of genome synteny at each *CYC* locus between core eudicot representatives (rosid and asterid) showed that gene content and order was generally well conserved for all three loci (Figure S3). In particular, genome synteny in these regions was very high between *Vitis vinifera* and *Prunus persica* (Figure S3), two rosid species which are believed to not have undergone further whole genome duplications after the putative genome triplication event γ prior to the divergence of rosids and asterids [42,43,71]. One exception is the rearrangement of the *HAC2*-*LINC1* complex which appeared translocated and in a reverse orientation at the *CYC2* locus in *V. vinifera*. By contrast *Arabidopsis thaliana* has fewer genes in common with the other rosid species, with between 24.14% and 38.48% of the full set of syntenic genes detected over the four core eudicot species (Table 1). Up to three other regions of the *A. thaliana* genome shared several genes syntenic with the *CYC* loci, but were missing *CYC*-like genes (see Figure S4). For *CYC1* two regions were identified on chromosome 1 (around AT1G4900 and AT1G73950); for *CYC2* three regions were identified, on chromosome 3 (AT3G02500) and chromosome 5 (AT5G16030 and AT5G38660); for *CYC3* three regions were identified, on chromosome 1 (AT1G25682 and AT1G13250) and chromosome 3 (AT3G25700) (Figure S4). In the asterid *Solanum* *lycopersicum* , collinearity with the rosid genomes was found to be generally extensive (with ~69% of the set of syntenic genes from the four core eudicot species found at the *CYC1* and *CYC3* loci for the A copies (Table 1)), although for the *CYC2* locus, no collinearity was detected in the region upstream of the *CYC* gene. This was also found to be the case in potato (*Solanum tuberosum*) and therefore does not seem an artifact of genome assembly (data not shown). Two regions on chromosome 2 (near Solyc02g085730.2 and Solyc02g063030.2) were identified as collinear with the upstream portion of the *CYC2* locus, indicative of translocation of these fragments. In addition, within each *CYC* clade, gene content was found to be more similar to the rosid loci for one of the two *S* *. lycopersicum* loci (Table 1).

**Table 1:** Percentage of syntenic genes present at the *CYC* loci in basal and core eudicots.

Species/copy no.	CYC1	CYC2	CYC3
*A. thaliana*	38.46	24.14	33.33
*P. persica*	92.31	93.10	94.44
*V. vinifera*	69.23	89.66	91.67
*S. lycopersicum*/A	69.23	31.03	69.44
*S. lycopersicum*/B	26.92	20.69	16.67
*S.* lycopersicum/A+B	76.92	34.48	77.78
*N. nucifera*/1	80.77	72.41	75.00
*N. nucifera*/2	65.38	72.41	61.11
*N.* nucifera/1+2	**88.46**	**89.66**	**77.78**
*A. coerulea*/1	50.00	41.38	61.11
*A. coerulea*/2	53.85	58.62	41.67
*A.* coerulea/1+2	**80.77**	**79.31**	**83.33**

Percentage of genes present for a given genomic fragment (regardless of location or orientation) from the list of genes in Figure S2. Combination of gene content from the two basal eudicot species (in bold) show that these are equally similar to the *CYC1*, *CYC2* and *CYC3* loci.

**Figure 3:**
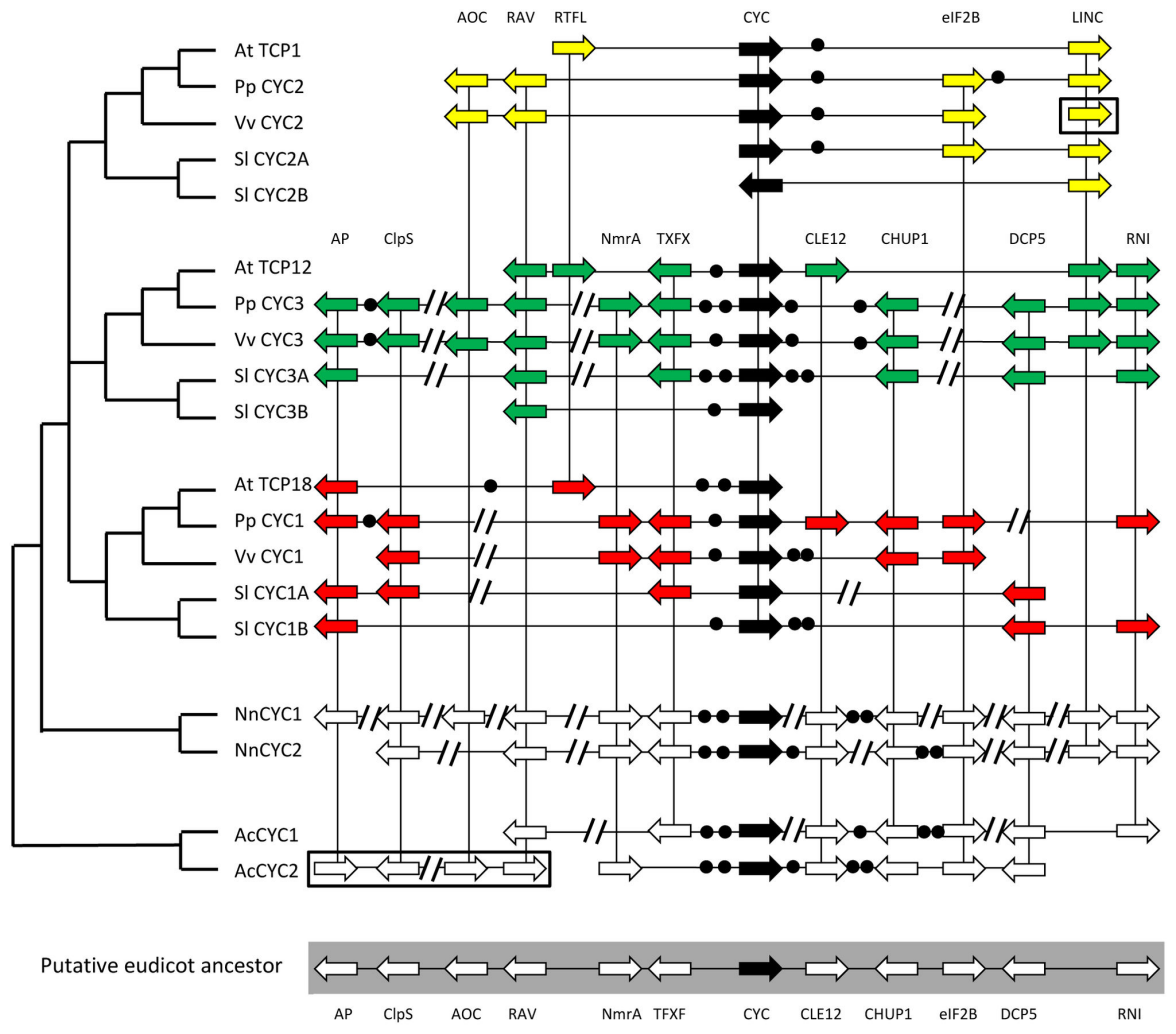
Synteny of duplicated genes at the *CYC* loci in core and basal eudicots. The phylogenetic tree to the left shows the relationship of the TCP ECE genes from *Arabidopsis thaliana*, *Prunus persica*, *Vitis vinifera*, *Solanum* *lycopersicum* *,* *Nelumbo* *nucifera* and *Aquilegia* *coerulea* . Abbreviated names of collinear genes are given above (full names given in Table S4), arrows show the presence and orientation of these genes, circles represent the number of non-syntenic genes (more than two contiguous non-syntenic genes are represented by parallel lines). Genes shared by at least two *CYC* loci are connected. Syntenic genes with a given core eudicot *CYC* locus are shown in the two *AcCYC* loci in *A* *. coerulea* . Boxed arrows show genes at the *V. vinifera CYC2* locus and the *AcCYC2* locus that are in a different location and orientation on the actual genomic fragment (shown in Figure S2 and Figure 4 respectively). A reconstructed gene map for the putative eudicot ancestor is shown, highlighted in gray.

**Figure 4:**
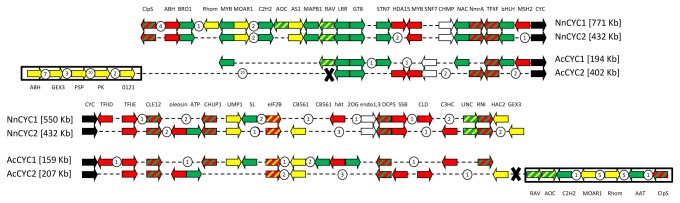
Synteny between the two *CYC* loci in *Nelumbo* *nucifera* and *Aquilegia* *coerulea* . Colored arrows represent genes which occur at the core eudicot *CYC* loci (*CYC1*: red, *CYC2*: yellow, *CYC3*: green), white arrows represent genes not found in the core eudicots sampled but shared by the two loci, circles represent non-syntenic genes. The crosses represent the putative translocation points in scaffold 26 in *A* *. coerulea* .

Although many collinear genes are specific to one of the three *CYC* loci, making them good markers for determining *CYC-*like gene homology within core eudicots, a number of genes are common to these regions (Figure 3). *RTFL* genes, from the *ROTUNDIFOLIA/DEVIL* gene family of signaling molecules, were found to be common to all three groups, but only in the *A. thaliana* genome. Although the association of *RTFL* and *CYC*-like genes was found in other species from currently available genomes in PGDD (e.g. *Cucumis* *sativa* , *Malus* x *domestica*, *Theobroma* *cacao* ), *RTFL* was not found in any syntenic block for the *CYC1* locus outside *A. thaliana*. Despite an absence of common genes between all three groups, a number of genes, in addition to *RTFL and CYC*, were found in common between pairs of groups: eight genes between *CYC1* and *CYC3* (*AP*-*ClpS*-*NmrA*-*TXFX*-*CLE12*-*CHUP1*-*DCP5*-*RNI*), three genes between *CYC2* and *CYC3* (*AOC*-*RAV*-*LINC*), and only one gene between *CYC1* and *CYC2* (*eiF2B*). Homologs of all these genes (but not *RTFL*) are present near the TCP ECE genes in one or both genomic fragments from the basal eudicots *N* *. nucifera* and *A* *. coerulea* , although *LINC* is absent in *A* *. coerulea* (Figure 3). In addition, orientation of these genes was conserved between basal and core eudicots, with the exception of certain genes (*AP*-*ClpS*-*AOC*-*RAV*) associated with *Aquilegia AcCYC2* where genomic rearrangements appeared to have taken place (see Figure 4). We therefore conservatively predict that the ancestral eudicot *CYC*–like locus had the following gene order: *AP*-*ClpS*-*AOC*-*RAV*-*NmrA*-*TFXF*-*CYC*-*CLE12*-*CHUP1*-*eIF2B*- *DCP5*- *RNI* (Figure 3). We cannot determine if *LINC* was present at that locus in the ancestor of all eudicots and lost at the *Aquilegia CYC* loci, or gained prior to the divergence of *N* *. nucifera* and core eudicots.

The two regions encompassing the TCP ECE paralogs in *A* *. coerulea* and *N* *. nucifera* showed extensive collinearity, however fractionation patterns differed between the two species (Figure 4). On the genomic fragments shown in Figure 4, the two regions in *N* *. nucifera* shared 29 genes out of 61, whereas in *A* *. coerulea* , comparable regions had 10 genes out of 45 in common. This corresponds to 47.5% and 22.2% of genes shared between the *N* *. nucifera* and *A* *. coerulea* fragments respectively, compared to ≤10% for comparable genomic segments in *V. vinifera*. Gene order and orientation were conserved between both basal eudicots, with the exception of a region at the *Aquilegia AcCYC2* locus (scaffold 26) where rearrangements (by translocation and inversion) can be inferred (Figure 4). Significantly, gene content at the *A* *. coerulea* and *N* *. nucifera* *CYC* loci was a mixture of most of the collinear genes from the three main core eudicot *CYC* loci; the set of genes in these basal eudicots was equally similar to each set of syntenic genes from the *CYC1*, *CYC2* and *CYC3* loci (Table 1, Figure S3).

## Discussion

TCP ECE genes have a complex evolutionary history not only in core eudicots but also in basal eudicots. By sampling extensively in the latter, we found that TCP ECE genes in basal eudicots have undergone independent duplications in all major lineages. For instance within Proteales, independent duplications appear have taken place in the ancestor of *N* *. nucifera* , in the ancestor of Platanaceae and Proteaceae, and in the ancestor of the Proteaceae subfamilies Grevilleoideae and Proteoideae. In Ranunculales, the evolutionary history of TCP ECE genes appears to consist of multiple duplication events, possibly one at the base of the order, and of independent gene losses, although this remains to be confirmed with a larger taxonomic sampling in this order, or by genomic comparisons when these become possible. This pattern of repeated duplication and paralog retention of TCP ECE genes in basal eudicots mirrors what has been described in core eudicots [49] and monocots [36,72]. In many cases, these duplications have been important in enabling new gene functions which have contributed to the elaboration of complex zygomorphic flowers (in the core eudicot *CYC2* clade e.g. [13,15,23,26]) but also in the monocot families Zingiberaceae [36] and Commelinaceae [37]).

Lineage-specific gene duplications have also been described in basal eudicots for floral developmental MADS-box genes. For instance within Ranunculales, *AP3* genes were found to have undergone two major duplications at the base of the order [73,74], *AP1* genes duplicated during or before the divergence of Ranunculales [75], and *AG* genes before the divergence of Ranunculaceae [76]. Independent duplications of MADS box genes, following a pattern similar to that of TCP ECE genes, have also been found in Platanaceae [73,77], Sabiaceae [78], Buxaceae [75,79,80] and Trochodendraceae [45]. Although it is not yet possible to determine whether MADS-box and TCP ECE duplication events coincided in different basal eudicot lineages, evidence from the *N* *. nucifera* genome, which has undergone a lineage-specific whole genome duplication (WGD), referred to as the λ event [81], suggests that the same polyploidy event may have resulted in the expansion of these different families of transcription factors. Indeed, similarly to what is described here for TCP ECE genes, the same two syntenic regions containing MADS-box genes can be found in *N* *. nucifera* for each *AP3*/*AG*/*AP1* paralog from *V. vinifera* (personal observation). It has been shown that some types of genes, especially transcription factors, are preferentially retained after large-scale duplication events [82]. Sub-functionalization and neo-functionalization appear to be the most common evolutionary pathways insuring paralog persistence over time [83]. In the case of transcription factors that regulate developmental processes, such as MADS-box and TCP ECE genes, spatio-temporal specialization and/or functional evolution of duplicates may result in an increased complexity of gene interactions, possibly enabling the emergence of novel phenotypes. The persistence of TCP and MADS-box gene duplicates has been considered significant for the evolution of core eudicot and monocot flower morphology (e.g. [37,49]). This persistence is also observed in early-diverging eudicot lineages, raising the hypothesis that it may contribute to the large morphological diversity observed among these taxa.

WGDs characterize the history of angiosperm lineages [84-86] and have been a major factor of gene family expansion. Local gene duplications (such as tandem duplications or distant transposition) have also contributed to the expansion of gene families (reviewed in [87]), including certain type I MADS-box genes (reviewed in [88]). In the case of the TCP ECE gene family, synteny analyses show that in both basal and core eudicots, expansion was driven by the duplication of large genomic segments. The pattern of collinearity between asterids and rosids at each of the three *CYC* loci suggests common ancestry from a single large fragment containing the *CYC* gene. Although we cannot completely rule out by phylogenetic means that one duplication (resulting in the *CYC1* and the ancestor of the *CYC2*/*CYC3* clades) occurred prior to the divergence of the eudicots, in the context of what is known about core eudicot genome evolution [41-45], and of the TCP ECE gene phylogeny inferred here from additional characters which shows that the three *CYC* clades form a monophyletic group (with genes from late-diverging basal eudicots), it is likely that these three loci arose from the γ genome triplication event. If the *CYC1* locus had originated from a duplication that had occurred before the divergence of eudicots, it may be expected that the *CYC2* and *CYC3* loci would show a higher level of synteny, which is not the case. The phylogeny of the TCP ECE genes does not unambiguously resolve the timing of the duplication events resulting in the *CYC1*, *CYC2*, *CYC3* clades, but suggests that these clades arose before the divergence of *Gunnera* and after the divergence of Proteales. The combined gene content of the syntenic fragments in *N* *. nucifera* were found to be equally similar to each core eudicot *CYC* locus, confirming that the duplications resulting in the *CYC1*, *CYC2*, *CYC3* clades post-date the divergence of Proteales. Large scale analysis of paralogous gene divergence in the late-diverging basal eudicot *Pachysandra* (Buxaceae) and early-diverging core eudicots ( *Gunnera* and *Vitis*) place the γ genome triplication after the divergence of Buxaceae and before the divergence of *Gunnera* [45]. For many gene families derived from this event, however, when taken individually, resolution for the timing of duplications along the deepest nodes within eudicots is lacking [44]. The nature of the polyploidy event possibly coincident with rapid speciation [89], as well as differential gene evolution, can all account for the lack of phylogenetic resolution [44]. Polytomies, as observed for the major core eudicot TCP ECE clades (e.g. in Figure 1), appear characteristic of gene families derived from the γ event. Similar results were found in recent phylogenetic reconstructions of MADS-box gene families where *Gunnera* and basal eudicots were extensively sampled [45]. Evidence from additional basal eudicot genomes, especially late-diverging species when these become available, will be crucial for understanding the evolutionary history of TCP ECE genes prior to the divergence of the core eudicots.

Both TCP ECE phylogeny and synteny analyses suggest that in the basal eudicots *A* *. coerulea* and *N* *. nucifera* , the large-scale duplication events occurred independently from each other and from the core eudicots. Within both species, synteny is higher between the two genomic fragments containing TCP ECE genes than between similar fragments within γ-derived genomes such as that of *V. vinifera*, suggesting these duplications may be more recent than the duplications that gave rise to the three core eudicot *CYC* loci. The λ WGD of *N* *. nucifera* is predicted to have taken place at the Cretaceous-Tertiary boundary around 65 million years ago (MYA) [81]. By contrast, the γ triplication event is estimated to have taken place ~120 MYA [45].

Synteny analysis between paralogous *CYC* loci highlights the complexity and diversity of genome evolution. Differential gene loss (fractionation) and genomic re-organization are evident between eudicot lineages. For instance, *A. thaliana*, which has undergone two additional WGDs (α and β) after the divergence of the core eudicots [43], displays extensive gene loss at all three *CYC* loci, compared to rosids such as *V. vinifera* [46] and *P. persica* [71] which have not undergone further duplications. Similar patterns have been described at the MADS-box *PI* and *AP3* loci [90]. Although three to four syntenic regions were identified in *A. thaliana* for each *CYC* locus (Figure S4), only one TCP ECE copy is found in each group; these WGDs have therefore not contributed to the expansion of TCP ECE genes in this species. In tomato, where a recent hexaploidization has been described [91], two copies are found in each *CYC* clade. In this species, the pattern of collinear gene retention does not appear equal between fragments, indicating biased fractionation [92]. Variability in gene loss/retention is also evident between the core eudicot *CYC1, CYC2, CYC3* clades, the number of syntenic genes differing between pairs of loci. In *A* *. coerulea* , genomic reorganization appears to have primarily occurred at one locus (*AcCYC2*). Synteny is well conserved in *N* *. nucifera* , which is consistent with the remarkably low evolutionary rate observed in this genome [81].

Basal eudicot genomes provide essential information for understanding the genomic events pre-dating the core eudicot radiation, and for reconstructing the ancestral eudicot-wide genome. We can predict, as a minimal hypothesis, that the ancestral eudicot locus contained the genes common to the duplicated core eudicot *CYC* loci, as has been predicted for B and C-class MADS-box genes [90]. Additionally, we find that the *CYC* loci in *A* *. coerulea* and *N* *. nucifera* contain a combination of most of the genes present at the three core eudicot loci. This suggests that, in addition, the ancestral locus might have comprised all of these genes beyond the minimal set we hypothesized, and that fractionation resulted in the pattern of gene distribution observed in core eudicots.

## Conclusions

The recently available genomic resources from two basal eudicots provide new insights into the evolutionary history of the TCP ECE genes at a crucial point in angiosperm diversification. As additional genomes become available, it will be possible to ascertain the origin of paralogous TCP ECE genes in other species. Regardless of their origin, retention of paralogs in all major basal eudicot lineages, where floral morphology is very diverse, is intriguing and suggests that these transcription factors may have the potential for a wide range of functions in basal eudicots, possibly through their role in the control of cell proliferation [52].

## Supporting Information

Figure S1 Angiosperm phylogeny (redrawn from the Angiosperm Phylogeny Website http://www.mobot.org/mobot/research/apweb/), showing the relationship of the basal eudicots sampled and *Gunnera* *tinctoria* ; new sequences were obtained from species in bold.
(PDF)

Figure S2 Reconciled tree of 21 eudicot TCP ECE sequences from 8 species.
(PDF)

Figure S3 Detailed synteny at the *CYC2*, *CYC3* and *CYC1* loci from *Arabidopsis thaliana*, *Prunus persica*, *Vitis vinifera*, *Solanum* *lycopersicum* ; the presence of homologous genes is shown for the basal eudicots *Nelumbo* *nucifera* and *Aquilegia* *coerulea* .
(PDF)

Figure S4 Synteny at the *CYC1*, *CYC2* and *CYC3* loci within the *Arabidopsis thaliana* genome.
(PDF)

Table S1 Primer sequences and combinations.
(XLSX)

Table S2 Sequence accession number from GenBank and the CoGe platform.
(XLSX)

Table S3 Summary of statistical tests of tree topology from the simplified eudicot data set.
(DOC)

Table S4 **Full names of collinear genes at the eudicot *CYC* loci.**
(XLSX)
