# Supplementary material for: Combining Phylogenetic and Syntenic Analyses for Understanding the Evolution of TCP ECE Genes in Eudicots
Source: PLoS One. 2013 Sep 3;8(9):e74803. doi: 10.1371/journal.pone.0074803 (PMC3760840; doi:10.1371/journal.pone.0074803)
Supplement: Figure S1 — Angiosperm phylogeny (redrawn from the Angiosperm Phylogeny Website http://www.mobot.org/mobot/research/apweb/), showing the relationship of the basal eudicots sampled and Gunnera tinctoria ; new sequences were obtained from species in bold. (PDF) [file pone.0074803.s001.pdf]

Figure S1. Angiosperm phylogeny (redrawn from the Angiosperm Phylogeny Website http://www.mobot.org/mobot/research/apweb/), showing the relationship of the basal eudicots sampled and *Gunnera tinctoria*; new sequences were obtained from species in bold. Clades marked by * have weak support. Provenance of samples is as follows: *C. laurifolius* Lyon Botanic Garden/011272; *N. domestica* Launay Botanical Park/001027; *E. alpinum* Launay Botanical Park/0011321; *A. quinata* Launay Botanical Park/000996; *C. agrestis* The Biodiversity of the Hengduan Mountains Project/31692; *M. myriantha* Strasbourg Botanic Garden; *N. nucifera* Lyon Botanic Garden/027144; *P. orientalis* Campus of Orsay University; *L. cordifolium* Roscoff Exotic Garden; *G. rosmarinifolia* Kerisnel Nursery, France; *B. sempervirens* Launay Botanical Park/006101; *T. sinense* Launay Botanical Park/002949; *G. tinctoria* Launay Botanical Park/001226.

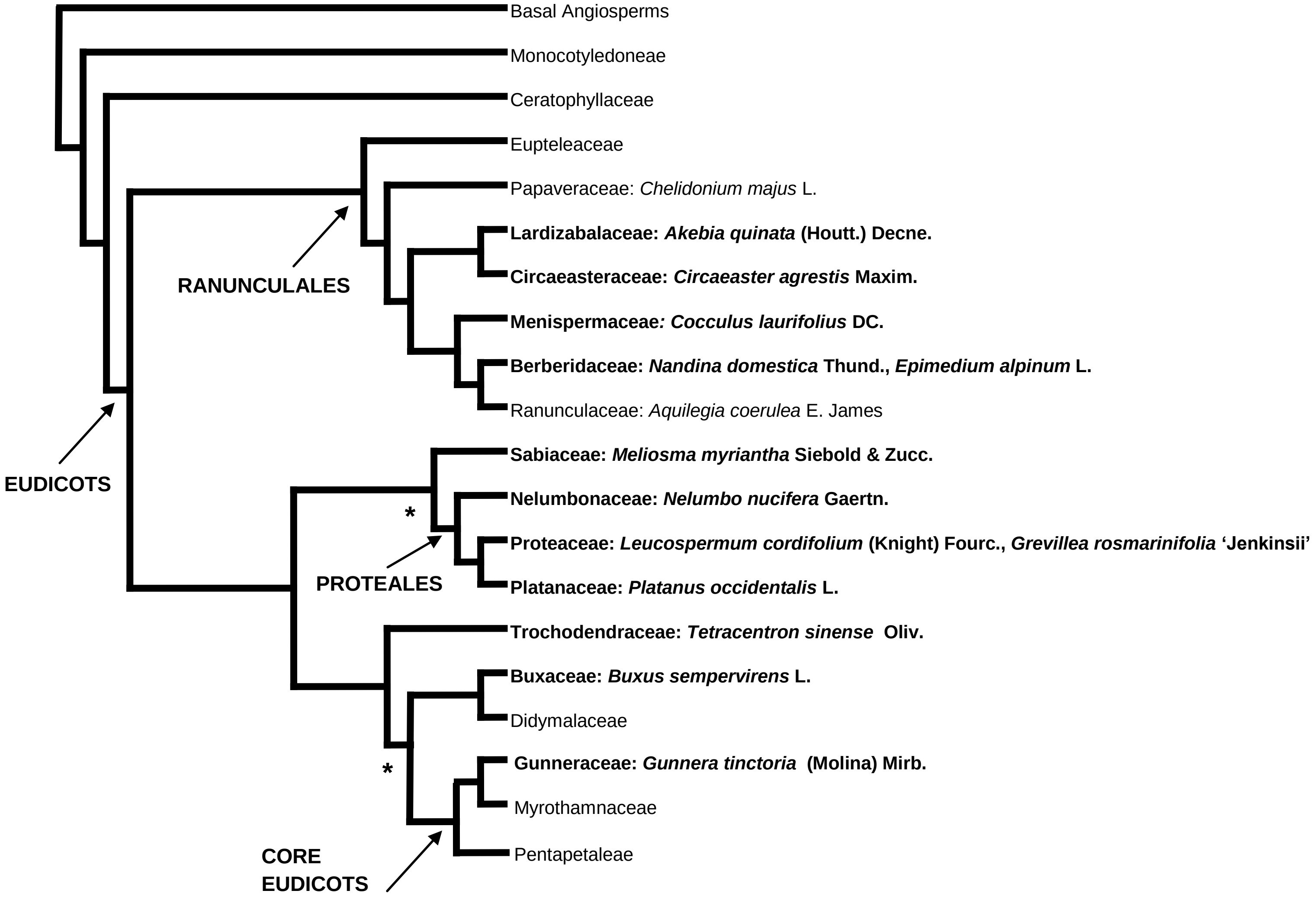

Basal Angiosperms
Monocotyledoneae
Ceratophyllaceae
Eupteleaceae
Papaveraceae: *Chelidonium majus* L.
Lardizabalaceae: *Akebia quinata* (Houtt.) Decne.
Circaeasteraceae: *Circaeaster agrestis* Maxim.
Menispermaceae*: Cocculus laurifolius* DC.
Berberidaceae: *Nandina domestica* Thund., *Epimedium alpinum* L.
Ranunculaceae: *Aquilegia coerulea* E. James
Sabiaceae: *Meliosma myriantha* Siebold & Zucc.
Nelumbonaceae: *Nelumbo nucifera* Gaertn.
Proteaceae: *Leucospermum cordifolium* (Knight) Fourc., *Grevillea rosmarinifolia* 'Jenkinsii'
Platanaceae: *Platanus occidentalis* L.
Trochodendraceae: *Tetracentron sinense* Oliv.
Buxaceae: *Buxus sempervirens* L.
Didymalaceae
Gunneraceae: *Gunnera tinctoria* (Molina) Mirb.
Myrothamnaceae
Pentapetaleae
RANUNCULALES
EUDICOTS
PROTEALES
CORE EUDICOTS
*
*
