# Supplementary material for: Combining Phylogenetic and Syntenic Analyses for Understanding the Evolution of TCP ECE Genes in Eudicots
Source: PLoS One. 2013 Sep 3;8(9):e74803. doi: 10.1371/journal.pone.0074803 (PMC3760840; doi:10.1371/journal.pone.0074803)

Figure S2. Reconciled tree of 21 eudicot TCP ECE sequences from 8 species. The gene tree was obtained by ML analysis (with PhyML) of a nucleotide matrix (363 aligned characters); branches were collapsed at <80% SH support value. Nodes with D indicate inferred duplication events.

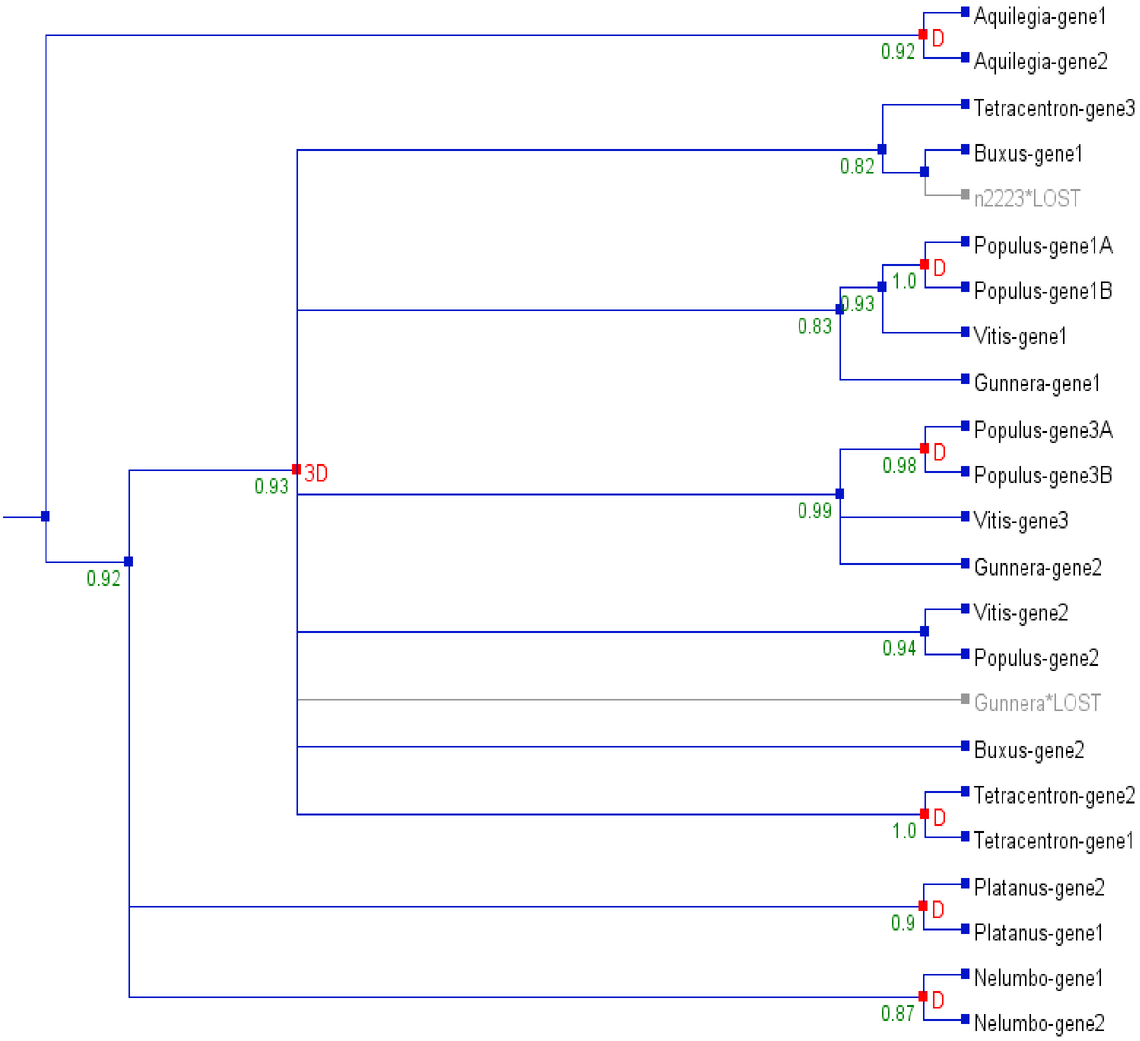

Supplement: Figure S2 — Reconciled tree of 21 eudicot TCP ECE sequences from 8 species. (PDF) [file pone.0074803.s002.pdf]
