# Supplementary material for: Combining Phylogenetic and Syntenic Analyses for Understanding the Evolution of TCP ECE Genes in Eudicots
Source: PLoS One. 2013 Sep 3;8(9):e74803. doi: 10.1371/journal.pone.0074803 (PMC3760840; doi:10.1371/journal.pone.0074803)
Supplement: Figure S3 — Detailed synteny at the CYC2, CYC3 and CYC1 loci from Arabidopsis thaliana, Prunus persica, Vitis vinifera, Solanum lycopersicum ; the presence of homologous genes is shown for the basal eudicots Nelumbo nucifera and Aquilegia coerulea . (PDF) [file pone.0074803.s003.pdf]

Figure S3. Detailed synteny at the *CYC2*, *CYC3* and *CYC1* loci from *Arabidopsis thaliana*, *Prunus persica*, *Vitis vinifera*, *Solanum lycopersicum*; the presence of homologous genes is shown for the basal eudicots *Nelumbo nucifera* and *Aquilegia coerulea*. Gene name is given above (full names given in Table S4), arrows show the presence and orientation of collinear genes, circles represent the number of non-syntenic genes. Genes shared by at least two *CYC* loci are connected. Boxed arrows are in a different location and/or orientation to that found for most species; for the *CYC2* locus in *V. vinifera*, the *HAC2-LINC1* complex is translocated; a number of gene blocks at the *AcCYC2* locus are in a different location and orientation with respect to that of eudicots, the actual gene order and orientation are shown in Figure 4.

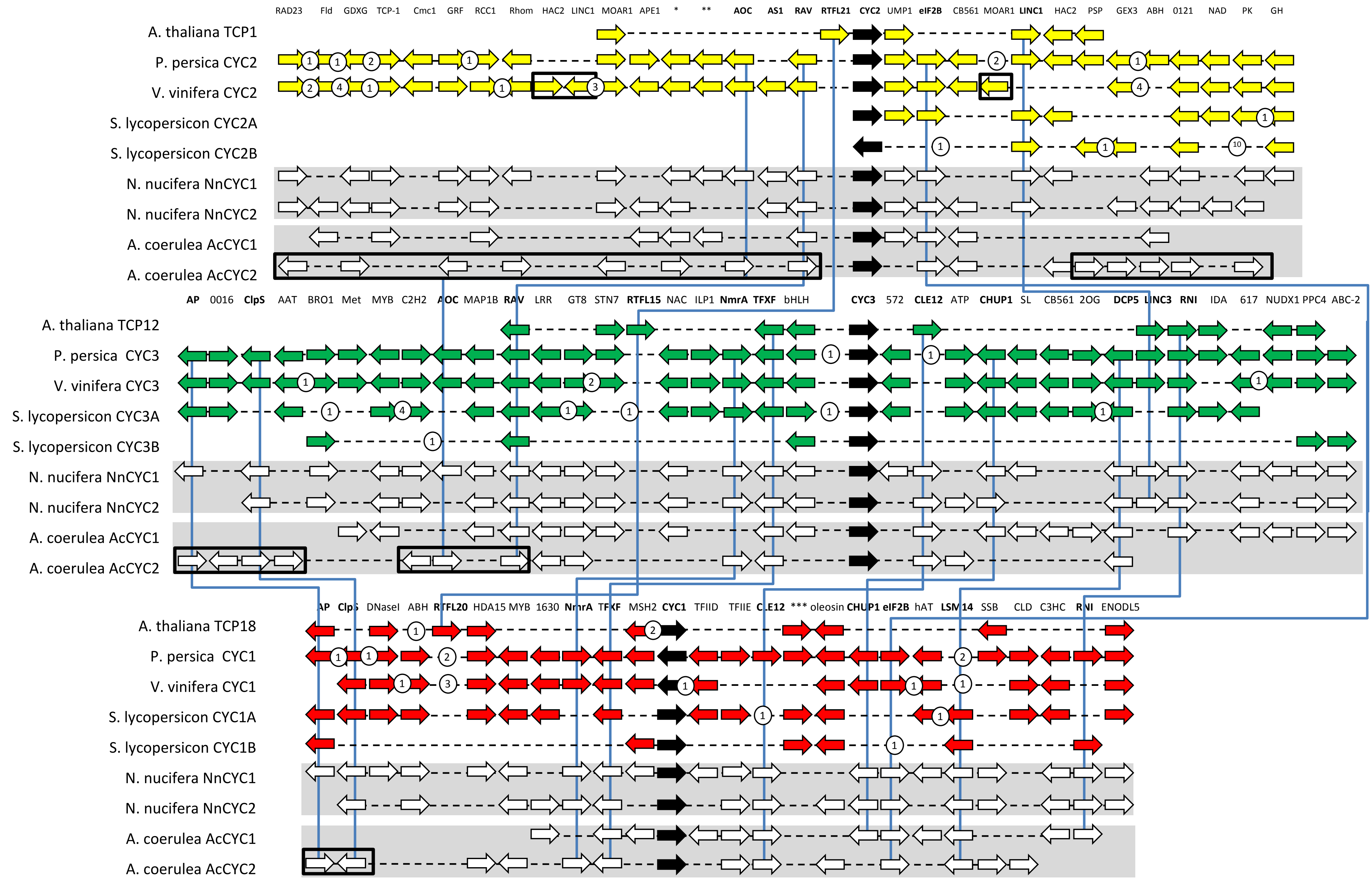
RAD23 Fld GDXG TCP-1 Cmc1 GRF RCC1 Rhom HAC2 LINC1 MOAR1 APE1 * ** AOC AS1 RAV RTFL21 CYC2 UMP1 eIF2B CB561 MOAR1 LINC1 HAC2 PSP GEX3 ABH 0121 NAD PK GH
A. thaliana TCP1
P. persica CYC2
V. vinifera CYC2
S. lycopersicon CYC2A
S. lycopersicon CYC2B
N. nucifera NnCYC1
N. nucifera NnCYC2
A. coerulea AcCYC1
A. coerulea AcCYC2
AP 0016 ClpS AAT BRO1 Met MYB C2H2 AOC MAP1B RAV LRR GT8 STN7 RTFL15 NAC ILP1 NmrA TFXF bHLH CYC3 572 CLE12 ATP CHUP1 SL CB561 2OG DCP5 LINC3 RNI IDA 617 NUDX1 PPC4 ABC-2
A. thaliana TCP12
P. persica CYC3
V. vinifera CYC3
S. lycopersicon CYC3A
S. lycopersicon CYC3B
N. nucifera NnCYC1
N. nucifera NnCYC2
A. coerulea AcCYC1
A. coerulea AcCYC2
AP ClpS DNaseI ABH RTFL20 HDA15 MYB 1630 NmrA TFXF MSH2 CYC1 TFIID TFIIE CLE12 *** oleosin CHUP1 eIF2B hAT LSM14 SSB CLD C3HC RNI ENODL5
A. thaliana TCP18
P. persica CYC1
V. vinifera CYC1
S. lycopersicon CYC1A
S. lycopersicon CYC1B
N. nucifera NnCYC1
N. nucifera NnCYC2
A. coerulea AcCYC1
A. coerulea AcCYC2
