# Supplementary material for: Combining Phylogenetic and Syntenic Analyses for Understanding the Evolution of TCP ECE Genes in Eudicots
Source: PLoS One. 2013 Sep 3;8(9):e74803. doi: 10.1371/journal.pone.0074803 (PMC3760840; doi:10.1371/journal.pone.0074803)
Supplement: Figure S4 — Synteny at the CYC1, CYC2 and CYC3 loci within the Arabidopsis thaliana genome. (PDF) [file pone.0074803.s004.pdf]

Figure S4. Synteny at the CYC1, CYC2 and CYC3 loci within the *Arabidopsis thaliana* genome. Syntenic fragments lack the TCP ECE gene (in green), and have genes which are missing from the *A. thaliana* CYC loci that are present at the CYC loci of other core eudicot species (all genes listed here). White arrows show the genes which are identified by number on the left. Boxed arrows show a reversed orientation for *LINC3-RNI* paralogues on the fragment containing AT1G13250, and an inversion (reversed orientation and order (*GH-CB*) compared to other core eudicots) for *CB-GH* on the fragment containing AT5G16030.

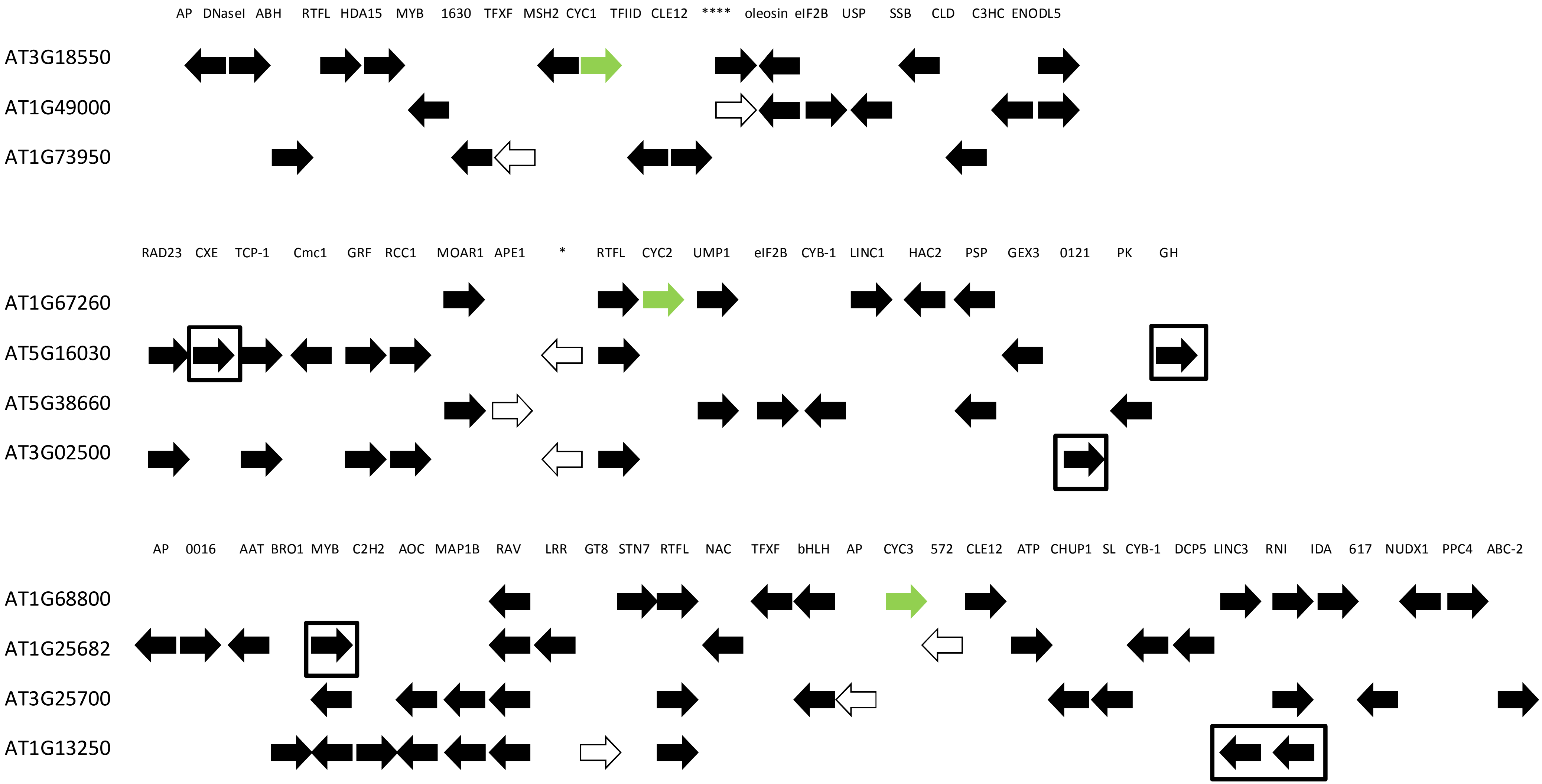
AP DNaseI ABH RTFL HDA15 MYB 1630 TFXF MSH2 CYC1 TFIID CLE12 **** oleosin eIF2B USP SSB CLD C3HC ENODL5
AT3G18550
AT1G49000
AT1G73950
RAD23 CXE TCP-1 Cmc1 GRF RCC1 MOAR1 APE1 * RTFL CYC2 UMP1 eIF2B CYB-1 LINC1 HAC2 PSP GEX3 0121 PK GH
AT1G67260
AT5G16030
AT5G38660
AT3G02500
AP 0016 AAT BRO1 MYB C2H2 AOC MAP1B RAV LRR GT8 STN7 RTFL NAC TFXF bHLH AP CYC3 572 CLE12 ATP CHUP1 SL CYB-1 DCP5 LINC3 RNI IDA 617 NUDX1 PPC4 ABC-2
AT1G68800
AT1G25682
AT3G25700
AT1G13250
