# Supplementary material for: Combining Phylogenetic and Syntenic Analyses for Understanding the Evolution of TCP ECE Genes in Eudicots
Source: PLoS One. 2013 Sep 3;8(9):e74803. doi: 10.1371/journal.pone.0074803 (PMC3760840; doi:10.1371/journal.pone.0074803)
Supplement: Table S3 — Summary of statistical tests of tree topology from the simplified eudicot data set. (DOC) [file pone.0074803.s007.doc]

**Table S3. Summary of statistical tests of tree topology from the simplified eudicot data set (see Material and Methods).** Loglikelihood for each tree were obtained with baseml (PAML v4.4). The Approximately Unbiased test and the weighted Shimodaira and Hasegawa test were carried out to assess the probabilities of the candidate trees to reflect the actual history.

| **Data set and topology tested (ranked by –log likelihood values)** | **-LnL**  **(DLi)** | **AU**  **P value** | **WSH**  **P value** |
| --- | --- | --- | --- |
| *Actual tree* | *- 4465.562* | 0.808 | 0.840 |
| CYC1 at the base | - 4.5 | 0.238 | 0.371 |
| CYC2 at the base | - 8.5 | 0.023 | 0.138 |
| CYC3 at the base | - 8.5 | 0.023 | 0.138 |
